# Persistent inguinal seroma managed with sprinkling of talcum powder: a case report

**DOI:** 10.1186/1752-1947-6-391

**Published:** 2012-11-21

**Authors:** Javier Lopez-Monclus, Miguel Angel Garcia-Ureña, Luis Alberto Blázquez, Daniel Adolfo Melero, Carmen Jiménez-Ceinos

**Affiliations:** 1 General Surgery Department, Henares Hospital, Avenida Marie Curie, s/n, 28822, Coslada, Madrid, Spain

## Abstract

**Introduction:**

We present a new method to treat recurrent seromas, which is based on our experience with a patient who had recurrent groin seroma and was treated successfully with a sprinkling of talcum powder in the seroma cavity.

**Case presentation:**

A 67-year-old Caucasian man with a suprapubic recurrent right groin hernia underwent inguinal hernioplasty with a polypropylene plug. Three days later the patient presented with a right groin fluctuating mass beneath the surgical wound with no signs of infection, and was discharged after seroma aspiration. After 23 days of increasing drainage, the seroma cavity was thoroughly dried with clean gauze swabs, and four g of sterilized dry talcum powder was sprinkled into the seroma cavity with a five-cc syringe. A compressive dressing was placed, and the patient was discharged. One week after the sprinkling of talcum powder, the surgical wound was almost closed with only minimal oozing from the drainage incision. The patient did not report any adverse effects. Two weeks later, the wound was fully healed.

**Conclusion:**

Talcum powder sprinkling could be an effective, quick, and safe method for the treatment of inguinal seromas after inguinal hernioplasty when conservative management has failed. Nevertheless, larger series are needed before assessing this technique as the treatment of choice.

## Introduction

Hernioplasty with prosthetic mesh is currently the treatment of choice for groin hernia, with lower recurrence rates than classical herniorrhaphies. Nevertheless, the use of prosthetic meshes is associated with postoperative complications such as increased rates of seroma and hematoma formation, chronic inflammation, infection, chronic pain and mesh migration [1].

A seroma is defined as a clinically identifiable collection of serous fluid in any tissue, potential space, or cavity after an operation. Seroma etiology remains unknown, but it seems to be due to a local inflammatory response to a mechanical injury incurred by tissue aggression during surgery and the presence of foreign bodies [2]. The use of drainages does not decrease the frequency of seroma formation [3], and a direct relationship exists between the amount of mesh in contact with subcutaneous tissue and the incidence of seroma [4]. Percutaneous or surgical drainage is required when a seroma becomes symptomatic, and this procedure is associated with a risk of infection. When a seroma persists despite successive drainages, it becomes a difficult-to-solve problem and an important impairment to the patient’s quality of life.

We present a case of recurrent groin seroma after an inguinal hernioplasty that was treated successfully with a sprinkling of talcum powder in the seroma cavity.

## Case presentation

A 67-year-old Caucasian man with a suprapubic recurrent right groin hernia after a Lichtenstein repair underwent a second inguinal exploration under local anesthesia, where the presence of suprapubic hernia recurrence was observed. A hernioplasty was carried out in which a polypropylene plug was placed in the recurrence point. No drain was left in place, and the patient was discharged the same day.

Three days later the patient arrived at the emergency room with a fluctuating mass located in the right groin below the surgical wound with no signs of infection, and he was discharged after seroma aspiration. Cultures of the aspirated fluid tested negative for bacterial growth.

On the 7th day after surgery, 50mL of seroma was aspirated again in the emergency room. On the 10th day after surgery, a one cm open drainage was carried out, evacuating 100cc of uncomplicated seroma, and a stoma bag was left in place to collect the drained fluid. From the 10th until the 22nd day after the surgery, a daily amount of between 100cc and 150cc of fluid was collected.

On the 23rd day, the seroma cavity was thoroughly dried with clean gauze swabs and four g of sterilized dry talcum powder (STERITALC® F4, Novatech, France) was sprinkled into the seroma cavity with a five-cc syringe. A compressive dressing was placed, and the patient was discharged.

During the next two days a local inflammatory response took place, with good pain control with standard analgesia, and a decreasing drainage of 50cc and 20cc respectively. One week after the talcum powder sprinkling, the surgical wound was almost closed with minimal oozing from the drainage incision. The patient did not report any adverse effects. Two weeks later, the wound was fully healed.

One year after surgery, the wound does not present any complications, there are no hernia recurrence signs, and the chronic pain has improved.

## Discussion

Seroma is a frequent complication after open repair of inguinal hernia, with a variable incidence reported by different groups due to it being underreported. Most seromas are asymptomatic and inconspicuous on inspection, and diagnosis is based on the clinical finding of a palpable fluid collection in the subcutaneous tissue.

Most seromas resolve spontaneously without any intervention. Park *et al*. [5] suggest that a seroma should be considered a complication only if it persisted for more than six weeks, presents continuous growth, or becomes symptomatic. If an underlying complication is suspected, such as infection or recurrence, then groin ultrasonography is the initial technique to confirm the nature of the swelling [6].

Despite its benign appearance, seroma persistence can become a major problem for patients, impairing their quality of life during the weeks until its complete resolution.

Nowadays there is no consensus on the management of symptomatic seroma: it varies from percutaneous aspiration to surgical drainage or the instillation of sclerosing substances.

Percutaneous seroma aspiration is the most widely used technique for symptomatic seroma management. This technique of repeated needle aspiration and mild application of external pressure was first described in 1971 [7], but it is associated with a higher risk of seroma infection and a high recurrence rate [8,9].

A more aggressive 3-trocar laparoscopic approach was described by Lehr and Schuricht [10] for treatment of persistent seromas after laparoscopic postincisional hernia repair. The technique described consists of evacuating both the serous fluid and the fibrinous debris followed by argon beam scarification of the seroma cavity lining. When seroma develops a thick surrounding capsule then it is considered a cystic seroma, and capsule removal might be the only curative option [11].

From our point of view, a persistent seroma could be extrapolated to the clinical scenario of a chronic pleural effusion: a cavity with a persisting exudative surface. Talcum powder was first used in 1935 to produce pleurodesis before carrying out a lobectomy. After this report, intrapleural talcum powder application has been demonstrated to be one of the most effective, simplest, and with the highest cost-benefit ratio, procedures for the treatment of recurrent pleural effusions. Talcum powder induces a strong fibrotic reaction in the pleural cavity due to the activation of polymorphonuclear neutrophils, interleukin 8 and fibroblast growth factor [12]. Complications related to talcum powder pleurodesis are not frequent; the most common adverse effect is pyrexia secondary to the inflammatory process, and major systemic complications are exceptional.

In our patient, talcum powder administration was easy and safe, with no complications and an outstanding result. The only complaint was a mild burning sensation in the groin area, and a local inflammatory response manifested as local redness that lasted 48 hours. Neither local nor systemic side effects have been described in the publications describing the use of talcum powder in abdominal wall surgery.

Nevertheless, the idea of using talcum powder as a treatment of symptomatic seromas it is not original to our group. In 1993, Coons *et al*. [13] published an experimental study in dogs comparing seroma formation in two groups after dissecting the latissimus dorsi muscle and applying talcum powder in one of them. They concluded that talc poudrage was clearly effective at minimizing seroma formation after the disection of musculocutaneous flaps. This article would open the unexplored field of talc poudrage to prevent seroma formation after inguinal or incisional hernioplasty.

In 2006, Saeb-Parsy *et al*. [14] described the application of talcum powder in an 8-month recurrent chronic seroma after breast surgery. They sprinkled four g of talcum powder inside the seroma cavity, and 10 weeks after its application the wound was completely healed.

Most recently, Klima *et al*. [15] have published the use of prophylactic subcutaneous talcum powder in incisional hernia repair, with a significant reduction of seroma formation and less wound infections and hernia recurrence. This report would support the safety of talc poudrage in abdominal wall surgery.

In fact, in 1983 an isolated article preconized the use of tetracycline sclerotherapy (the other most extended pleurodesis technique) for the treatment of persistent seromas; the physical basis of this variant would be the same as in the talcum powder technique described.

## Conclusion

Talcum powder sprinkling could be an effective, quick, and safe method for the treatment of inguinal seromas after inguinal surgery. It could be an alternative to conservative management in seromas refractories to conservative management. The technique described in this case can be easily reproduced and presents an extremely high cost-benefit ratio. From a theoretical point of view, its application as a prophylactic therapy for seroma formation should be investigated. Nevertheless, this is an isolated case report and larger series and randomized clinical trials are needed before assessing this technique as a treatment of choice.

## Consent

Written informed consent was obtained from the patient for publication of this manuscript. A copy of the written consent is available for review by the Editor-in-Chief of this journal.

## Competing interests

The authors declare that they have no competing of interests.

## Authors’ contribution

MAG-U conceived the idea for the article. LAB and DAM performed the clinical data review. CJ-C and JL-M were the major contributors in writing the manuscript. All authors read and approved the final manuscript.
